# Pediatric autoimmune neuropsychiatric disorders associated with streptococcal infections (PANDAS) in a two-year-old

**Published:** 2024-05

**Authors:** Hal Steven Farkas, Ellen Werber Leschek

**Affiliations:** 1 College of Osteopathic Medicine, Nova Southeastern University, Fort Lauderdale, FL, USA.; 2 National Institute of Diabetes and Digestive and Kidney Diseases, National Institutes of Health, Bethesda, MD, USA.

**Keywords:** Pediatric Autoimmune Neuropsychiatric Disorders Associated with Streptococcal infections (PANDAS), Acute Rheumatic Fever (ARF), Acute Post-Streptococcal Glomerulonephritis (APSGN), Asymptomatic Group A Beta-Hemolytic Streptococcal infections (GABHS)

## Abstract

Streptococcal pharyngitis testing and treatment is not routinely recommended in children under the age of 3 because of the unlikely occurrence of infection and negligible risk of serious complications. However, streptococcal pharyngitis and its resulting complications are not uncommon in this age group and can have serious consequences. We report a case of Pediatric Autoimmune Neuropsychiatric Disorders Associated with Streptococcal infections in a 2-year-old with streptococcal pharyngitis. Testing and treatment for streptococcal pharyngitis should be strongly considered when there is evidence of infection and/or an immune-mediated streptococcal complication to prevent and/or decrease the severity of short- and long-term complications.

## Introduction/Background

Current guidance published by prominent sources states that children under the age of 3 are unlikely to develop Group A Beta-Hemolytic Streptococcal (GABHS) pharyngitis and its complications [1–5]. In fact, GABHS pharyngitis and its complications do occur in this age group, so the threshold for testing and treatment should be low when there is any indication of infection.

The association between GABHS pharyngitis and serious immune-mediated complications has been well established. Among these important resulting illnesses are Acute Rheumatic Fever (ARF), Acute Post-Streptococcal Glomerulonephritis (APSGN) and Pediatric Autoimmune Neuropsychiatric Disorders Associated with Streptococcal Infections (PANDAS). While these serious complications are more common in children older than 3, they can occur at very young ages as well. Timely treatment of GABHS pharyngitis and prevention of subsequent infections can reduce or prevent the risk of short- and long-term complications [6–9]. Tani et al. evaluated 541 cases of rheumatic fever seen at the University of Utah between 1985 and 2000 and found that approximately 5% of affected children were younger than 5 years at presentation and 0.9% were under 3 [10]. Marshall et al.found that of 415 confirmed APSGN cases in the Northern Territory of Australia, 6.7% were children under 3 [11]. Of the first 50 cases of PANDAS reported by Swedo et al, 4% were under 3 years of age [12].

Immune-mediated GABHS pharyngitis complications can lead to devastating short- and long-term effects. ARF frequently damages heart valves and is a significant contributor to cardiovascular morbidity and mortality [13]. Chronic complications of APSGN include hypertension, nephrotic syndrome and chronic renal failure [14–16]. PANDAS involves inflammation of the basal ganglia, which may irreversibly alter this area of the brain, resulting in chronic chorea, tics, obsessive-compulsive disorder and/or other basal-ganglia-related signs and symptoms [17–19].

We present a case of PANDAS in a 2-year-old with no signs or symptoms of GABHS pharyngitis. If published streptococcal pharyngitis guidance had been followed, it is likely that this child would not have been assessed for a GABHS infection, and treatment would not have been rendered, putting this child at risk for prolonged short-term effects of immune-mediated symptoms as well as long-term complications. PANDAS and other GABHS pharyngitis immune-mediated complications occur in children under the age of 3, and guidelines should be updated to ensure that providers diagnose and treat GABHS pharyngitis in children under 3 years of age.

## Case Presentation

A healthy 2.3-year-old male child acutely developed severe eye blinking and vocal tics. No other symptoms were noted. Physician evaluation revealed only the tics reported by the parents. Pharyngeal swab, requested by the mother, showed a positive rapid strep test, and a 10-day course of amoxicillin was initiated. The tics slowly improved and resolved by completion of the antibiotic regimen. However, within days of the GABHS pharyngitis diagnosis, he developed sensory avoidance symptoms and hyperacusis, including refusal to wear a jacket, insistence that tags be removed from clothing items and frequent covering of ears to variable levels of noise. These symptoms persisted. GABHS antibodies were obtained 1 day after antibiotic completion. Antideoxyribonuclease-B antibody (Anti-DNase B) was elevated at 336 U/mL (normal range 0–77 U/mL) and Antistreptolysin O antibody (ASO) was negative at <20.0 IU/mL (normal range 0.0–200.0 IU/mL). Anti-DNase B was repeated 9 days later and was 165 U/mL (normal range 0–77 U/mL).

Mild transient eye-blinking tics lasting 1–2 days occurred at both 4 and 6 weeks after amoxicillin completion. An acute viral upper respiratory infection was present during each of these episodes. Rapid strep assessment and GABHS pharyngeal culture were negative on both occasions. One week after the second brief episode, streptococcal antibodies were again measured. Anti-DNase B was 195 U/mL (normal range 0–77 U/mL) and ASO was <20.0 IU/mL (normal range 0.0–200.0 IU/mL).

At 2.6 years of age, severe eye blinking tics abruptly returned. They were more intense and frequent than those that occurred with the initial documented GABHS pharyngitis. The only symptom, aside from the tics and persistent sensory avoidance and hyperacusis issues, was irritability. Physician evaluation revealed only the tics reported by the parents, and pharyngeal swab showed a positive rapid strep test. A 10-day course of cephalexin was initiated (amoxicillin allergy was diagnosed on the final day of treatment for the first GABHS pharyngitis) as well as ibuprofen 3 times daily for its anti-inflammatory properties. Ibuprofen was continued for 3 weeks. The irritability stopped several days after initiation of antibiotic and ibuprofen, and the tics and sensory avoidance issues resolved approximately 4 weeks after antibiotic completion. The hyperacusis persisted.

Family history was significant for PANDAS in the mother (diagnosed at the National Institutes of Health [NIH]), two of the mother’s maternal first cousins (diagnosed at the NIH) and a second cousin once removed on the maternal side. In addition, the mother’s maternal grandmother had APSGN at 2 years of age, resulting in hospitalization for 2 months.

Based on the above clinical and laboratory data as well as the family history, a diagnosis of PANDAS was made. Prophylactic azithromycin was initiated.

GABHS pharyngitis and its immune-mediated complications must be considered in children under the age of 3 years. This patient did not have any signs or symptoms of GABHS pharyngitis but presented with a known immune-mediated complication of GABHS pharyngitis. GABHS testing revealed that he had asymptomatic GABHS pharyngitis. Reluctance to test children under 3 years of age for GABHS pharyngitis, as is recommended by many trusted sources, could have delayed diagnosis and treatment of this individual, resulting in chronic tics and/or other basal-ganglia-related signs and symptoms.

## Discussion/Conclusion

Testing for GABHS pharyngitis is not encouraged under the age of 3 years because it is thought that children of this age are unlikely to become infected or develop complications [1–5]. While it is less common for GABHS pharyngitis to occur in this age group, it does occur and can be associated with significant short- and long-term complications. Among these are serious immune-mediated diseases, including ARF, APSGN and PANDAS.

ARF, one autoimmune complication of GABHS pharyngitis, occurs when streptococcal M-proteins mimic cardiac myosin, causing the immune system to damage heart tissue and valves [20]. Acute illness due to ARF can be severe and may include arthritis, fever, breathlessness and edema from heart failure. Sydenham’s chorea, another manifestation of ARF, occurs in up to 25% of ARF-affected individuals. Sydenham’s chorea also results from molecular mimicry. Anti-neuronal antibodies form in response to GABHS infection and target the basal ganglia of the brain, causing involuntary choreiform movements and other motor symptoms, tics and neuropsychiatric issues (anxiety, depression, obsessions and compulsions and attention deficit disorder) [21]. Long-term effects of ARF can include irreversible valvular scarring, which is referred to as Rheumatic Heart Disease (RHD). Regurgitation from the damaged valve(s) may lead to chronic fatigue, swollen extremities, and arrhythmia and can result in potentially fatal tertiary complications such as heart failure, bacterial endocarditis or ruptured heart valves [22]. If cardiac valve damage is significant enough, valve replacement may be the only treatment option. RHD can occur in children following a single case of ARF or after multiple episodes [6]. The incidence of ARF is highest in children 5–14 years old, but it does occur in children as young as 2- to 3-years-old [6]. According to Miyake et al, in children under 21, the ARF incidence rate is 14.8 cases per 100,000 children hospitalized with an average age of 10 years [23].

APSGN, another immune mediated complication of GABHS pharyngitis, is thought to result from the formation of immune complexes that deposit in the renal tissue [24]. Acute illness generally involves some combination of hematuria, hypertension, oliguria, edema, increased serum creatinine and nephrotic syndrome. These issues completely resolve in about 90% of children who develop APSGN following a GABHS infection, but some go on to develop chronic renal conditions, including nephrotic syndrome, hypertension, left ventricular failure and chronic renal failure [15]. APSGN is most prevalent in children of early school age but can occur at any age [25]. One study demonstrated that 4% of children with GABHS pharyngitis developed APSGN and found that children under 6.5 years of age with GABHS infections are at an increased risk of developing APSGN compared to older individuals [26]. Marshall et al.estimate that the incidence of APSGN is 94.3 cases per 100,000 in children under 15 years of age [11]. According to the World Health Organization (WHO), more than 400,000 children are diagnosed with APSGN worldwide each year and at least 1% of those affected die each year due to the acute inflammation or its associated long-term complications [27].

PANDAS is a more recently described immune-mediated complication of GABHS pharyngitis. Like ARF and APSGN, molecular mimicry is implicated in the etiology of PANDAS. It is thought that antibodies formed in response to GABHS infection cross-react with tissues of the basal ganglia [28]. This can result in the neurologic and neuropsychiatric signs and symptoms associated with PANDAS, including tics, abnormal movements, Obsessive Compulsive Disorder (OCD) and anxiety. PANDAS is a clinical diagnosis that relies on the following diagnostic criteria: (1) presence of OCD, tics or both, (2) pediatric onset of symptoms (generally 3- to 12-years-old and prepubertal), (3) episodic course of symptom severity with dramatic ups and downs, (4) association with GABHS pharyngitis, (5) neurological abnormalities and (6) abrupt onset or worsening of symptoms. Other findings associated with PANDAS include the new onset of hyperactivity, inattention, fidgeting, separation anxiety, mood changes (irritability, sadness, emotional lability), trouble sleeping, enuresis, frequent daytime urination, changes in motor skills and joint pains [12,29]. PANDAS signs and symptoms onset very suddenly during or following GABHS pharyngitis and last from several weeks to months or longer. If the affected individual contracts another case of GABHS pharyngitis, symptoms worsen suddenly and are often more severe and of longer duration than the previous episode [29]. While most children fully recover from PANDAS, chronic neuropsychiatric symptoms occur in some people, especially those with recurrent or untreated GABHS infections. In cases that go untreated and/or when there are many recurrences, tics, OCD and other signs and symptoms are more likely to continue into adulthood [18]. Risk for the development of long-term sequelae in PANDAS is not surprising since MRI demonstrates inflammation and enlargement of the basal ganglia and nearby structures in individuals with PANDAS [30,31]. Lepri et al.reported that relapse of neurological symptoms was seen in 45% of PANDAS patients over a long period. They also found that antibiotic prophylaxis substantially reduced neurologic symptoms in PANDAS patients over a 7-year period, further stressing the need for early diagnosis and treatment to reduce the risk of progression to disabling chronic neurologic sequelae [19]. The incidence of PANDAS is poorly understood because this disease was first described less than 30 years ago. However, Dr. Susan Swedo, who first characterized PANDAS and studied the disease extensively, estimates that as many as 25% of children diagnosed with OCD and tic disorders have PANDAS [32].

In a study of pediatric patients with new GABHS infections, 65% were found to have generated an antibody response to one or more GABHS antigens despite being completely asymptomatic [33]. A longitudinal study evaluated the relationship between movements/behaviors and GABHS pharyngitis in 693 elementary school-aged children. In addition to showing an association between motor/behavior changes and positive GABHS throat cultures, the study demonstrated a seasonal (fall) peak in GABHS infections that was likely driven by the detection and treatment of many asymptomatic children [34]. Information published by the Centers for Disease Control and Prevention in 2022 notes that in approximately one-third of individuals who develop ARF, this complication was preceded by a subclinical GABHS infection (or infections) [35]. These and other studies demonstrate that many children do not exhibit any symptoms during episodes of GABHS pharyngitis but are nonetheless at risk for the development of immune-mediated GABHS complications.

The patient described in this report clearly meets the diagnostic criteria for PANDAS. He presented with acute onset of tics in conjunction with GABHS pharyngitis documented by positive throat swab and positive Anti-DNAse B titer. His tics resolved in approximately 10 days and abruptly recurred in a more severe fashion 3 months later during another documented GABHS pharyngitis. The second PANDAS episode lasted about 4 weeks. Between the GABHS infections, there were two extremely mild, transient tic episodes during viral upper respiratory infections (GABHS pharyngeal cultures were negative), which is not uncommon in children with PANDAS, particularly when it is soon after a PANDAS episode. The negative pharyngeal cultures between his two classic PANDAS episodes provides support for a clear relationship between his severe PANDAS symptomatology and GABHS pharyngitis. While he is younger than 3 years of age, the arbitrary lower limit of the age range for PANDAS, of the first 50 PANDAS patients described by Swedo et al., 4% were under 3.12. The patient reported here had an episodic course of symptom severity, with dramatic ups and downs, meeting another diagnostic criterion for PANDAS. Lastly, he had neuropsychiatric signs and symptoms associated with both GABHS pharyngitis infections, including sensory avoidance issues and hyperacusis. In addition, he had a strong family history of immune-mediated disease due to GABHS pharyngitis, and while not a diagnostic criterion, positive family history is a known PANDAS risk factor [36,37].

It is well documented that GABHS pharyngitis occurs in children under 3 years of age and can be associated with profound short- and long-term complications. While a number of prominent sources discourage GABHS pharyngitis testing in children under 3 years of age [1–5], diagnosis of this infection must be followed by an immediate course of the appropriate antibiotic, regardless of age, to prevent or decrease the severity of immune-mediated complications (ARF, ASPGN, PANDAS) [6,38,39]. If the GABHS pharyngitis is associated with ARF or PANDAS, follow-up with ongoing GABHS antibiotic prophylaxis is indicated to prevent exacerbations and/or recurrences [8,40]. Because many cases of GABHS pharyngitis are asymptomatic [33–35], evidence of GABHS immune-mediated complications without symptoms of GABHS pharyngitis should be pursued. Rapid strep testing, throat culture and/or streptococcal antibody titers should be performed to determine whether there is an active or recent GABHS infection. This is important to the attenuation and/or prevention or short- and long-term complications.

Testing for GABHS pharyngitis is discouraged for children under the age of 3 years in online guidance posted by Johns Hopkins, Cleveland Clinic, Mayo Clinic and the Centers for Disease Control and Prevention [1–4]. The reasons stated include that children of this age are unlikely to become infected or develop complications. While GABHS pharyngitis is less likely to occur in this age group, these infections absolutely do occur in children under 3 years of age and there is an association with significant short- and long-term complications in this age group. Among these are serious immune-mediated diseases, including ARF, APSGN and PANDAS. These serious complications are more common in children older than 3, but they can certainly occur in children younger than this arbitrary cutoff. For these reasons, guidance should be modified to recommend testing for GABHS pharyngitis in all children, regardless of age, who have signs and symptoms consistent with this infection and/or are experiencing an immune-mediated complication of GABHS infection (ARF, ASPGN, PANDAS). This is critical because these complications may lead to irreversible disease.

## Abbreviations:

Anti-DNAse B: Antideoxyribonuclease-B Antibody
APSGN: Acute Post-Streptococcal Glomerulonephritis
ARF: Acute Rheumatic Fever
ASO: Anti-Streptolysin O
GABHS: Group A Beta Hemolytic Streptococcal (infection)
OCD: Obsessive-Compulsive Disorder
PANDAS: Pediatric Autoimmune Neuropsychiatric Disorders Associated with Streptococcal Infections
RHD: Rheumatic Heart Disease
